# A Novel Non-Planar Bioprinting Methodology for Enhanced Surface Fidelity of the Cornea

**DOI:** 10.3390/bioengineering13060682

**Published:** 2026-06-12

**Authors:** Laura Pérez Sánchez, Hodei Gómez-Fernández, Maialen Zelaia Amilibia, Maria Basañez Elorrieta, Eva Larra Mateos, Alessandro Scandurra, José Luis Pedraz Muñoz, Denis Scaini, Camilo Cortés

**Affiliations:** 1 Digital Health and Biomedical Technologies, Vicomtech Foundation, Basque Research and Technology Alliance (BRTA), Donostia-San Sebastian, Spain; 2 AJL Ophthalmic, Ferdinand Zeppelin Kalea, 01510 Vitoria-Gasteiz, Spain; 3 NanoBioCel Research Group, Laboratory of Pharmacy and Pharmaceutical Technology, Department of Pharmacy and Food Science, Faculty of Pharmacy, University of the Basque Country (UPV/EHU), Paseo de la Universidad 7, 01006 Vitoria-Gasteiz, Spain; 4 BIOMAT Research Group, University of the Basque Country (UPV/EHU) Escuela de Ingeniería de Gipuzkoa, Plaza de Europa 1, 20018 Donostia-San Sebastian, Spain; 5 Makby, 20009 Donostia-San Sebastian, Spain; 6 Bioaraba, NanoBioCel Research Group, 01009 Vitoria-Gasteiz, Spain; 7 Networking Research Centre of Bioengineering, Biomaterials and Nanomedicine (CIBER-BBN), Institute of Health Carlos III, 28029 Madrid, Spain; 8 Joint Research Laboratory (JRL) on Bioprinting and Advanced Pharma Development, A Joined Venture of TECNALIA, Centro de Investigación Lascaray Ikergunea, Avenida Miguel de Unamuno, 01006 Vitoria-Gasteiz, Spain; 9 eHealth Group, Bioengineering Area, Biogipuzkoa Health Research Institute, 20014 Donostia-San Sebastian, Spain

**Keywords:** non-planar trajectories, surface parametrization, poisson mesh reconstruction, corneal tissue engineering, morphological fidelity

## Abstract

Traditional 3D bioprinting of corneal constructs relies on planar slicing, which often results in a significant stairstep effect and the loss of anatomical curvature. Curvilinear layering has emerged as a promising alternative to address these limitations. The presented methodology, based on non-planar layer integration, ensures a smoother surface finish. The model’s surface is identified via vertex normals and reconstructed using the Poisson method. Finally, surface parametrization is applied to generate spatially curved trajectories. To validate the algorithm, corneal constructs were printed using a planar and the proposed non-planar approach. Quantitative evaluation of micro-Computed Tomography data revealed that the non-planar approach achieved significantly higher morphological fidelity, successfully replicating the intended parabolic profile of the human cornea. Furthermore, the non-planar constructs demonstrated adequate functional performance, characterized by high optical transparency. Thereby, the feasibility of printing non-planar layers using the proposed novel approach is successfully demonstrated. Furthermore, the comparative analysis confirms the method’s potential for corneal biofabrication when compared to traditional planar methods.

## 1. Introduction

Additive Manufacturing (AM) is an innovative technology that enables the fabrication of a 3D model through the continuous accumulation of material in layers [1,2]. Today, tissues and organs produced using 3D bioprinters show potential for improving surgical outcomes [3] and treating various injuries [4,5,6] by enabling the fabrication of patient-specific implants that precisely match the injury geometry, mimic the structure and function of the target tissue, and incorporate autologous cells, thereby reducing the risk of immune rejection. Three-dimensional bioprinting uses AM to deposit biomaterials and cells with the aim of creating models that can mimic the architecture observed in organs and tissues [7,8,9]. During the regeneration process, scaffolds—supportive structures—play a critical role by acting as extracellular matrices, responsible for providing a permissive environment to encourage cell attachment, proliferation, and differentiation [10,11]. A scaffold should contain a porous structure [4] to facilitate nutrient transport to cells and to remove metabolic waste [10].

The complex architecture of the cornea—characterized by specifically organized collagen fibrils and distinct cellular layers—poses significant challenges for traditional tissue engineering. Three-dimensional bioprinting offers a promising solution by enabling the precise, layer-by-layer fabrication of corneal tissues that closely mimic the essential characteristics required for vision restoration and long-term graft success [12]. Though small, the cornea is remarkably complex; it is a convex, aspheric dome with a horizontal diameter of 11–12 mm and a vertical diameter of 9–11 mm [13]. Its thickness increases gradually from approximately 535–560 µm at the center to a periphery that is roughly 100 µm thicker [14]. This symmetric curvature, defined by an anterior radius of ~7.8 mm and a posterior radius of 6.5 mm, allows the cornea to contribute approximately two-thirds of the eye’s total refractive power [15,16]. Furthermore, its optical function is predicated on extreme transparency, exhibiting 80–94% light transmission at 450–600 nm and up to 98% at longer wavelengths [17].

In the field of corneal regeneration, Digital Light Processing (DLP) and extrusion bioprinting are the most widely used manufacturing methods [18]. DLP bioprinting enables fast fabrication and high resolution through the layer-by-layer photopolymerization of photosensitive bioinks, allowing entire layers to be solidified simultaneously [19]. However, when highly transparent bioinks—required to mimic the optical properties of the cornea—are used, light is not sufficiently attenuated along the z-axis. This results in uncontrolled light propagation beyond the intended curing plane, leading to overpolymerization or poorly defined crosslinking boundaries, and consequently a loss of resolution that limits the accurate fabrication of fine structures. To overcome this limitation, photoabsorbers such as tartrazine [20] have been introduced to restrict light penetration and improve printing fidelity. However, this strategy is not suitable for corneal applications, as these additives introduce unwanted coloration and reduce optical transparency [21,22].

In addition, the vat-based configuration of DLP systems results in significant material waste, since excess bioink—often containing costly primary cells—cannot be reused [23]. Finally, although DLP is generally considered cytocompatible, the intrinsic cytotoxicity of some photoinitiators and unreacted monomers, as well as light-induced oxidative stress, remains a limitation that can affect long-term cell viability and function [24].

In contrast, extrusion bioprinting stands out for its adaptability and manufacturing efficiency. This technique involves the continuous deposition of materials through a nozzle, driven by pneumatic or mechanical pressure, to build 3D structures. It is particularly versatile due to its capacity to process a wide range of bioink compositions and high-viscosity materials [12].

The most common approach for generating 3D-printed models is to divide the model into a series of planar layers [1,10]. A slicing plane is used to intersect the target model at different heights [10]. While a dome shape can be approximated by printing concentric rings or 3D spirals [25], standard planar slicing produces a stairstep error that increases with layer thickness and local slope [10]. These steep overhanging curvatures often lead to significant deviations from the design radius and potential structural collapse. Therefore, following the planar slicing methodology for reconstructing complex geometries, increasing the number of layers is necessary to reduce the stairstep effect [26], which, in turn, results in increased fabrication time [1]. Even with such improvements in vertical resolution, the discretized nature of planar layering does not guarantee that the stairstep effect disappears, particularly on high-curvature surfaces.

Moreover, when corneal constructs are biofabricated with hydrogel-based materials, the presence of the fragmented paths generated by slicing makes it arduous to construct structurally entangled hydrogel-based three-dimensional structures; consequently, constructs might have insufficient structural strength [10]. To overcome these limitations and accurately replicate the native corneal architecture, recent advancements suggest a transition from traditional planar slicing to curvature-conformal toolpath generation combined with specialized molds for extrusion bioprinting [12,27].

In recent years various methods have been implemented to reduce the stairstep effect and to improve the mechanical properties of the model [1,28]. Adaptive slicing, such as in the studies of Dolenc et al. [29] and Kulkarni et al. [30], focuses on improving the surface quality and shortening the printing time, by adapting the slice thickness depending on the local geometry [31,32,33,34,35,36]. Beyond adjusting layer thickness, a more fundamental shift involves moving away from flat layers entirely toward non-planar paths that stick to the geometry of the 3D model [37]. In this latest approach, movements along the z-axis are allowed to produce 3D curvatures, although the printhead must be considered to avoid collisions with the geometry [10]. Non-planar paths have been demonstrated to enhance the mechanical properties, the deposition continuity, and the layer adhesion of the target [38]. Furthermore, non-planar trajectories can reduce the biomaterial waste and improve the production rate of complex biomimetic shapes [28] by minimizing the number of underfilled regions [1].

There are several approaches to generate non-planar paths. Liao et al. [10] proposed a novel non-planar grid slicing process for printing irregularly shaped tissues with low aspect ratios. Etienne et al. [39] created an algorithm capable of deforming the model, slicing the deformed model with the traditional planar method to finally deform the generated paths back to the original space. Shan et al. [34] developed a method by utilizing isothermal surfaces in a heat transfer simulation, points with the same temperature level represented a natural curved layer. In Zhao et al. [4], Lian et al. [40] and Wang et al. [41] utilized approaches based on the generated point clouds after scanning lesions are used, path planning is obtained by projecting a pattern. In Zhao et al. [42], an optimization algorithm is presented that identifies optimal waypoints in the point clouds to avoid the distortion caused by projecting a 2D pattern onto a 3D surface. In the successive studies of Chakraborty et al. [43], Singamneni et al. [44], Jin et al. [45], Chen et al. [46], Alkadi et al. [47] and Zhao et al. [48], the drastic reduction in the stairstep effect has been implemented by generating and offsetting curved layers. In addition, approaches based on the decomposition of a model in parts to generate collisions free trajectories are present also in the literature [49,50,51,52].

Many existing algorithms are tailored for internal volume filling rather than the high-precision requirements of thin-film surface printing. Furthermore, while various methodologies have been proposed to overcome the limitations of planar layering in general Additive Manufacturing, there is a notable absence of frameworks specifically designed for the curvilinear geometries found in ocular tissues. Consequently, existing non-planar strategies remain unproven for such applications.

In response to these challenges, this study presents the following main contributions:

(a) To the best of our knowledge, this is the first study to address the applicability of non-planar trajectories for corneal biofabrication. This contribution establishes a dedicated framework for thin-film, curvilinear ocular geometries. Specifically, the proposed algorithm consists of the combination of the Poisson method and surface parametrization to generate non-planar trajectories. By leveraging the Poisson mesh reconstruction algorithm [53], a 3D surface is generated from a set of unorganized points. This reconstructed surface then serves as the foundation for a mesh parametrization algorithm [54], which is applied to compute and execute the non-planar path planning.

(b) The definition of a predesigned planar trajectory and its printing onto a non-planar surface, ensuring similarity with the use of mesh parametrization algorithm and bridging the gap between traditional 2D slicing and 3D curvilinear trajectory implementation.

(c) The proposed algorithm is evaluated through the printing of corneal constructs. By comparing the resulting morphology against traditional planar methods—which constitute an available (open-source software) baseline for this specific application as suggested in [25]—this study demonstrates a significant enhancement in surface fidelity and structural integrity, and adequate optical transparency values, highlighting the potential of this approach for corneal biofabrication.

## 2. Materials and Methods

The methodology developed for this study comprises five distinct stages: (1) the identification of the model’s surface geometry; (2) surface reconstruction; (3) the postprocessing of the surface; (4) the generation of non-planar trajectories via parametrization; and (5) the evaluation of the methodology through the printing of corneal constructs and the subsequent characterization of their optical transparency and morphological fidelity.

### 2.1. Identification of the Model’s Surface

The proposed methodology expects as input a 3D model in Stereolithography Interface Format (STL format) file. In this file, the model is represented as piecewise linear 3D surface (mesh), composed of a group of triangles called facets, which must comply with specific vertex-to-vertex and facet orientation rules [55]. The algorithm implemented in this study consists of detecting the surface by considering the z-component of the vertex normals in the given mesh. The vertices with a normal z-component value higher than a given threshold are selected as candidates to points belonging to the top layer surface.

### 2.2. Surface Reconstruction

**Figure 1:**
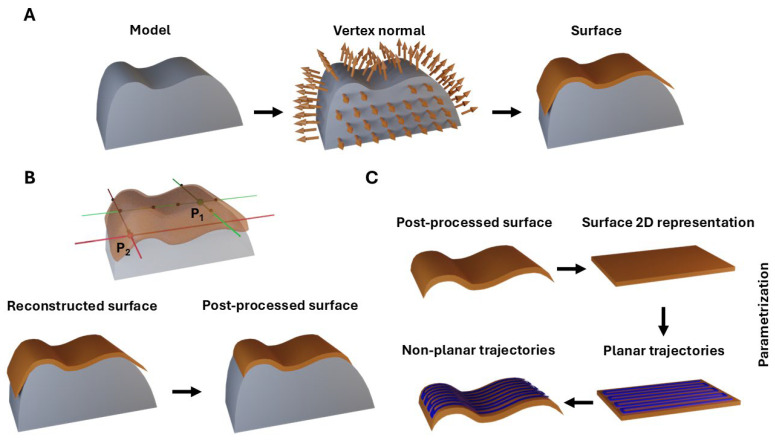
(**A**) Visualization of the surface points detection using vertex normals and surface reconstruction with the Poisson Surface Reconstruction method. (**B**) The surface is postprocessed according to the original surface of the model. (**C**) Representation of the process to generate non-planar trajectories: (i) the surface is mapped in a plane through parametrization; (ii) planar trajectories are computed in the 2D space; and (iii) non-planar trajectories are obtained when returning to the 3D space.

Given a set of 3D points with oriented normals, the Poisson Surface Reconstruction method [53] produces an extrapolated surface version of the top layer, trying to preserve the normals of the selected candidate points. The generated surface is an extrapolated surface similar to a blanket that covers the model (see Figure 1A).

### 2.3. Surface Postprocessing

The vertices of the surface mesh generated with Poisson method are mapped to a (XY) plane in the parameter space, corresponding to their planar domain [54]. Therefore, the surface mesh has a 2D representation.

The reconstructed surface mesh and its 2D representation are postprocessed according to the original surface of the model. The points of the surface mesh are modified; the raycasting test [56] is proposed to postprocess the surface mesh. In the raycasting test, rays are computed for each point in the X and Y direction. If a ray intersects an even number of times with the model in a given direction, it means that it is not contained in the original model and is removed. A representation of the raycasting test is observed in Figure 1B. The gray area represents the original model and the orange area the reconstructed surface. For each point of the reconstructed surface the raycasting test is performed. The *P*_1_ point (in green) should be preserved, whereas the *P*_2_ point (in red) should be removed. At the bottom, the resulting postprocessed surface can be observed. The removed points are eliminated from both the surface mesh and its 2D representation. After the postprocessing, the algorithm searches for scattered faces and removes them to avoid incongruences when generating the trajectories.

### 2.4. Generation of Non-Planar Trajectories

The 2D representation of the postprocessed surface mesh is used as a result of a traditional slicing; thus, planar trajectories are generated. To ensure high-fidelity mapping back to the 3D space, these trajectories are discretized into a series of dense coordinates for each individual stroke. This discretization is critical; by increasing the point density along each path, the subsequent non-planar trajectories more accurately conform to the curvilinear morphology of the model, preventing linear interpolation errors that would otherwise deviate from the intended curved surface (see Figure 1C).

To transform the planar trajectory into non-planar trajectory, the barycentric coordinates are used. Since the parametrization applied consists of an angle-preserving mapping, the barycentric coordinates permit an accurate interpolation. Each point *P* of the planar trajectory can be represented by three non-aligned points *P*_0_, *P*_1_ and *P*_2_. The numbers *b*_0_, *b*_1_ and *b*_2_ constitute the barycentric coordinates of a point *P* if the conditions in (1) and (2) are satisfied [57]:

(1) $$P=b_{0}P_{0}+b_{1}P_{1}+b_{2}P_{2},$$

(2) $$b_{0}+b_{1}+b_{2}=1.$$

Barycentric coordinates can be calculated in a simple manner from the points *P*_0_, *P*_1_ and *P*_2_ by defining *A*_0_ as the area of the triangle *PP*_1_*P*_2_, *A*_1_ as the area of the triangle *PP*_2_*P*_0_, and *A*_2_ as the area of the triangle *PP*_0_*P*_1_ (see (3)). So, the barycentric coordinates *b*_0_, *b*_1_ and *b*_2_ are

(3) $$b_{0}=\frac{A_{0}}{A_{0}+A_{1}+A_{2}},\;b_{1}=\frac{A_{1}}{A_{0}+A_{1}+A_{2}},\;b_{2}=\frac{A_{2}}{A_{0}+A_{1}+A_{2}}.$$

Barycentric relationships can be applied for 2D and 3D coordinates. Therefore, for each point of the planar trajectory, the facet of the surface mesh that contains the point is identified. Then, the areas are calculated using the corresponding facet of the 2D representation of the surface mesh to obtain the barycentric coordinates. The 3D vertices of the facet and the barycentric coordinates are substituted into (1) to obtain the point of the trajectory in the original space.

### 2.5. Experimental Evaluation

The evaluation of the proposed algorithm is conducted by performing a comparative analysis between traditional planar printing and the non-planar printing method introduced in this study. To validate the algorithm’s applicability and its ability to improve surface quality, both methods are used to print corneal constructs.

#### 2.5.1. CAD

**Figure 2:**
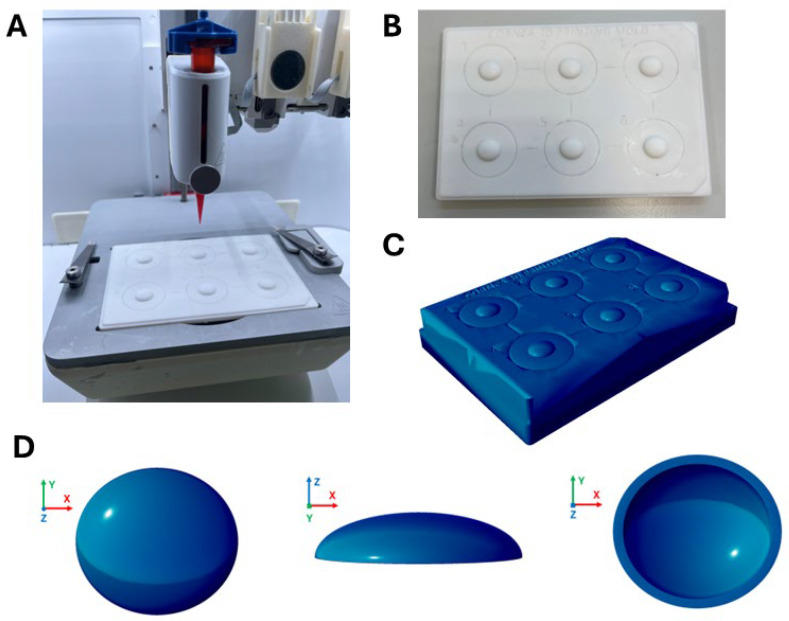
Bioprinting setup. (**A**) Overview of the BIO-X™ extrusion bioprinter with the printhead positioned over the (**B**) support fabricated via 3D SLA printing. (**C**) CAD base used for both planar and non-planar trajectory deposition. (**D**) The 3D corneal model. The design features a dome-shaped morphology based on human physiological parameters. Left to right: superior view, lateral view and inferior view.

Onshape software V1.174(PTC, Boston, MA, USA) is used to generate the STL file of the 3D model (see Figure 2D). The geometry is defined based on physiological parameters, including a dome-shaped morphology with a horizontal diameter of 11,460 μm and a vertical diameter of 10,380 μm, with a central thickness of around 500 μm gradually increasing towards the periphery [13,58]; the total height of the Computer-Aided Design (CAD) model is 2431 μm.

#### 2.5.2. Traditional Planar and Non-Planar Trajectories

The planar trajectory and G-code are generated in PrusaSlicer V2.9.0 (Prusa Research, Prague, Czech Republic) and then processed in Repetier Host software V2.3.1 (Hot-World GmbH & Co. KG, Willich, Germany). The trajectory is optimized to ensure the uniform deposition of the bioink across the construct, with a layer height of 0.15 mm and concentric perimeters and top layer, for maintaining structural integrity.

In contrast, the non-planar trajectory is generated with an in-house processor, following the Poisson-based parametrization approach detailed in Section 2.5.1, which allows the toolpath to conform to the curvilinear surface of the model. These complex coordinates are translated into machine-readable G-code instructions.

#### 2.5.3. Bioink

The bioink used corresponds to that developed under patent number P202530162 by AJL Ophthalmic S.A., consisting of a collagen-based hydrogel. This material is specifically designed for the fabrication of corneal scaffolds via 3D printing and bioprinting, owing to its transparency, biocompatibility, suturability, and ability to withstand the mechanical stresses of the cornea.

#### 2.5.4. Printing Process

The bioprinting of corneal constructs is performed using the 3D extrusion bioprinter BIO-X™ from CELLINK (Gothenburg, Sweden) (see Figure 2A). The bioink is loaded into a 30 mL cartridge with UV protection, equipped with a piston and a blunt 25G needle (0.25″, CELLINK).

To prevent the bioink from collapsing due to the steep overhanging curvature of the corneal dome, the constructs are printed onto a support fabricated via 3D SLA printing (see Figure 2B,C), using the Form 3 printer and the biocompatible BioMed White Resin V1, both supplied by Formlabs (Somerville, MA, USA). This support incorporates the specific 3D corneal geometries designed in the previous stage (see Figure 2D), allowing the bioink to be deposited directly onto the intended anatomical curvature.

For non-planar printing, a temperature of 30 °C and a pressure of 30 kPa is applied, using a speed of 17 mm/s. In planar printing, the same temperature of 30 °C is maintained, but with a higher pressure of 40 kPa to avoid gaps in the bioink, and a constant speed of 17 mm/s is used.

### 2.6. Quantitative Metrics

To quantify the proposed algorithm improvements, the performance is measured against a specific set of metrics:

#### 2.6.1. Transparency

The spectral transmittance of planar and non-planar corneal scaffolds is evaluated in the UV-A and visible spectral ranges. Measurements are performed using a Tecan Infinite M200 over a wavelength range of 300–700 nm, using Phosphate-Buffered Saline (PBS) as a negative control.

The percentage transmittance (%T) is calculated from the obtained sample absorbance values ($A_{sample}$) by applying the relationship derived from the Beer–Lambert law [59] and correcting against the negative control ($A_{C-}$) according to (4):

(4) $$T\left(\%\right)=10^{\left(2-\left(A_{sample}-A_{C-}\right)\right)}.$$

#### 2.6.2. Morphological Fidelity Analysis

Printed corneas are scanned and reconstructed using micro-Computed Tomography (micro-CT). This high-resolution imaging allows for a direct comparison between the original CAD model, Ground Truth (GT), and the actual printed geometry. The images obtained with micro-CT provide a clear visualization of the cornea’s curvature, showing the algorithm’s ability to maintain structural fidelity. The morphological fidelity is assessed through two distinct analytical approaches: global height statistical analysis and cross-sectional curvature profiling.

##### Micro-CT Acquisition and 3D Model Reconstruction

Scanning is performed using SkyScan 1172 micro-CT system V1.5 (Bruker microCT, Kontich, Belgium) with an X-ray source that operates at 48.0 kV and 167.0 µA. To preserve the structural integrity of the corneas, the scanning protocol is optimized to ensure hydration retention.

A rotation of 180° with a rotation step of 0.30° is performed to minimize the total time acquisition, as extending the scan would introduce significant artifacts. Protocol setup testing demonstrated that this 0.30° step, which yielded 600 projection images, provides sufficient spatial resolution without compromising the geometric integrity of the printed cornea. Furthermore, a large pixel size of 27.4 µm is selected to accelerate the scanning process. To maintain the high image quality, a frame averaging of four and a random movement setting of ten are utilized. This approach is critical to prevent the corneas from drying out or deforming during the radiation exposure, ensuring that the captured geometry represents the actual printed state. Following the scan, a series of high-resolution cross-sectional slices at regular intervals along the vertical axis of the cornea are computed with SkyScan’s NRecon software V1.7.4.2 (Bruker microCT, Kontich, Belgium), generating a stack of Digital Imaging and Communications in Medicine (DICOM) files. To minimize distortions, the reconstruction process incorporates a box asymmetry smoothing kernel, misalignment compensation, a ring artifact reduction value of two, and an 18% beam-hardening correction. These slices provide the foundational volumetric data for all subsequent morphological analyses.

##### Mesh Generation

For each printed cornea, a 3D model is obtained using the reconstructed DICOM files. The 2D slices are imported into a custom Python V3.13 environment. By stacking the slices, a 3D volumetric array is obtained and processed with a Gaussian filter to mitigate acquisition noise. Then, segmentation is performed to isolate the corneal construct from the background through a binarization process. By leveraging the significant grayscale intensity contrast between the deposited bioink and the surrounding environment in the micro-CT scans, a quasi-optimal threshold is determined to ensure precise volumetric separation of the printed geometry. Once segmented, the Marching Cubes algorithm is applied to the binary volume, generating a high-resolution triangular surface mesh. Since corneas are hydrated to avoid drying out, a Region of Interest (ROI) is defined to ensure the analysis focuses solely on the cornea. This step allows for the removal of base artifacts and, by applying a connectivity filter, ensures that only the cornea is extracted for final measurement.

##### Height Analysis

To assess the structural fidelity across both the planar and non-planar printing methods, the vertical accuracy of every printed cornea is systematically compared to the original CAD. This metric is computed by determining the bounds along the z-axis, the difference between the maximum and minimum z-coordinates for each construct. Box plots are selected for visualization to provide a comprehensive representation of the distribution, highlighting the reproducibility of the height measurements. To evaluate the statistical significance of the differences between methods, an independent two-sample t-test is performed.

##### Topographic Curvature Profiling

The morphological fidelity of the cornea is analyzed by examining the cross-sectional curvature profile. The developed algorithm identifies the vertex with the maximum height and performs a 2D slice along the coronal plane. To ensure the profile only represents the surface and remains free from artifacts, a sequential jump detector is utilized. The vertical distance between consecutive points along the lateral axis is analyzed; if a sudden drop is detected, the point is discarded as an artifact. This filtering step is necessary to eliminate discontinuities often generated during the 2D slicing of complex meshes, and it is applied consistently to both the printed samples and the original CAD reference. The remaining coordinates are then binned and interpolated onto a common lateral axis to allow for direct group comparisons. Beyond the overall shape of the constructs, the Area Under the Curve (AUC) is calculated for each method. The AUC serves as a metric for structural fidelity, quantifying the total cross-sectional area successfully reconstructed in relation to the GT.

## 3. Results and Discussion

### 3.1. Experimental Evaluation

**Figure 3:**
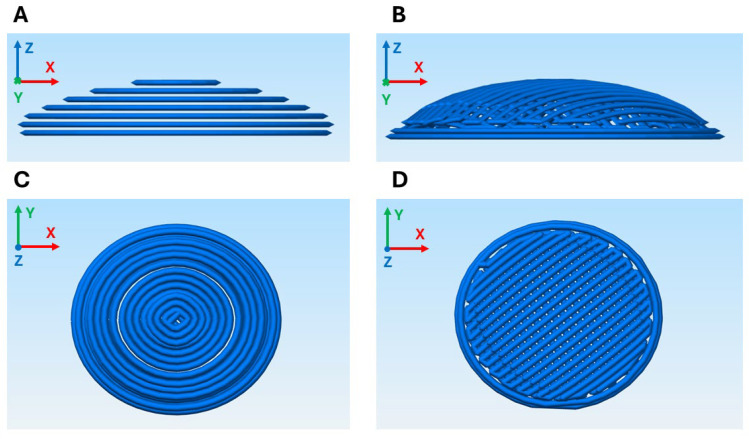
Representation of planar and non-planar trajectories for corneal model. (**A**) Lateral view of the planar trajectory, illustrating the discrete horizontal layers that result in a staircase effect. (**B**) Lateral view of the non-planar trajectory, showing the curvilinear trajectories that conform to the target geometry. (**C**) Superior view of the planar trajectory, highlighting the spiral pattern. (**D**) Superior view of the non-planar trajectory.

The generated trajectories for both methodologies are illustrated in Figure 3, where the staircase effect inherent to the planar methodology is clearly visible (see Figure 3A). This geometric approximation results in discrete, stepped layers that deviate from the smooth, natural profile of the target tissue. Regarding the non-planar trajectories, two layers were generated, performing an offset in the z-axis (see Figure 3B,D).

The algorithmic efficiency of the proposed non-planar method scales proportionally with the geometric complexity of the input mesh. A higher density of facets demands greater processing time for the Poisson Surface Reconstruction and barycentric coordinate interpolations. For the corneal geometry utilized in this study, which consisted of a total of 37,070 facets, the execution of the data processing and toolpath generation algorithms required approximately five minutes utilizing a conventional laptop computer (Intel Core i7-11800H, 2.30 GHz, 16 GB RAM). It is worth noting that this computational cost is incurred exclusively during the processing and trajectory calculation stage. Once computed, the non-planar G-code can be executed indefinitely for batch manufacturing, effectively decoupling processing time from physical production. It should be highlighted that no algorithmic or execution time optimization was attempted in this study, leaving substantial potential for future runtime improvements through code refactoring or parallelization.

**Figure 4:**
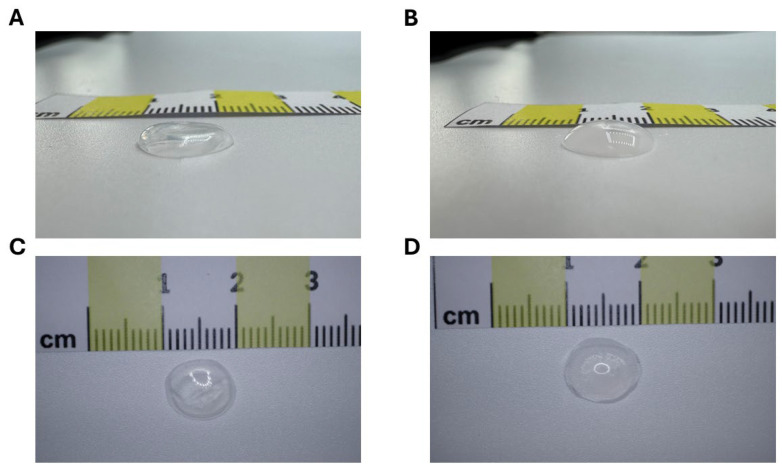
Visual comparison between planar (**A**,**C**) and non-planar (**B**,**D**) 3D-bioprinted corneal constructs. (**A**,**C**) Profile and superior view of a construct fabricated via planar layering, noticeable light scattering is observed. (**B**,**D**) Profile and superior view of a construct fabricated via the non-planar approach, light presents less distortion.

**Figure 5:**
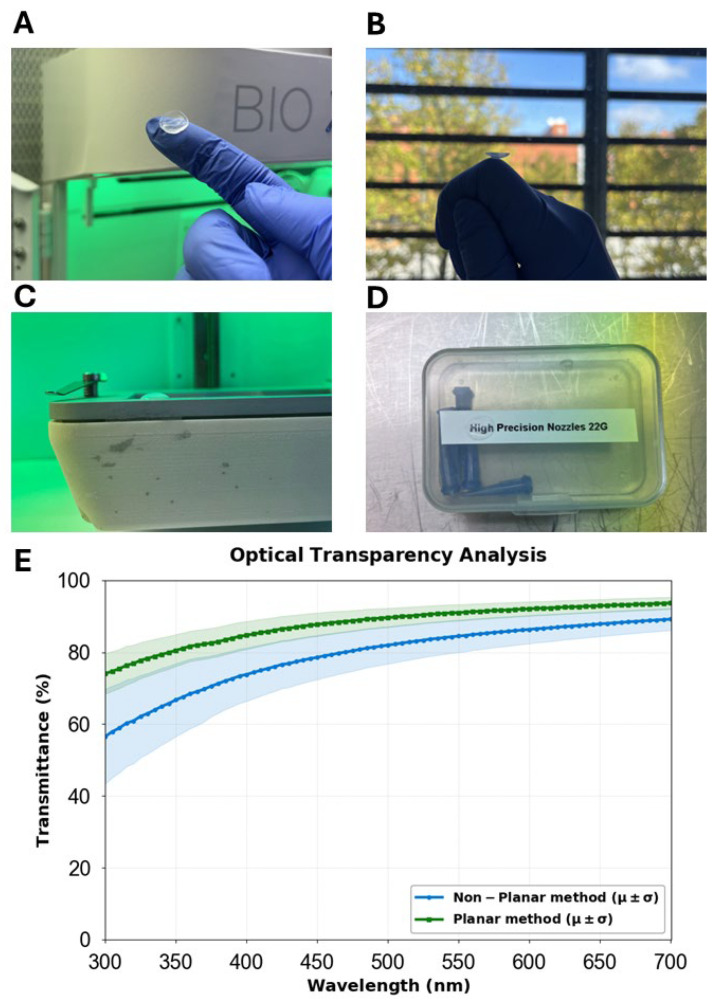
Corneal construct printed with the non-planar methodology. (**A**,**B**,**D**) Visualization of a corneal construct through distinct perspective views to observe the structural integrity and thin-surface geometry. (**C**) Top view of the cornea placed over text to qualitatively assess optical transparency and clarity of a non-planar construct. (**E**) Transparency analysis of biofabricated corneal constructs. The graph illustrates the percentage of light transmittance across the visible spectrum (300–700 nm) for both printing methodologies: (i) Planar (in green); (ii) non-planar (in blue). The solid lines represent the mean transmittance, while the shaded regions indicate the standard deviation.

To evaluate the repeatability and structural performance of the proposed algorithm, a total of 20 corneal constructs were bioprinted. Specifically, ten corneas were printed using the traditional planar methodology (see Figure 4A,C), while ten corneas were printed using the non-planar methodology (see Figure 4B,D and Figure 5A–D). This sample size allowed for a comparative statistical analysis of morphological fidelity and functional outcomes between the two trajectory strategies. Figure 4 presents a visual comparison between the resulting planar and non-planar bioprinted constructs. A preliminary visual inspection reveals that the non-planar constructs exhibit superior structural integrity; unlike the planar samples, which show signs of slumping or collapsing under their own weight, the non-planar constructs maintain a well-defined aspherical dome. This suggests that the curvature-conformal deposition not only improves surface fidelity but may also enhance the overall mechanical stability of the scaffold during the fabrication process. A qualitative assessment of the optical transparency and clarity of a non-planar cornea is illustrated in Figure 5D.

### 3.2. Quantitative Metrics

The following sections detail the quantitative metrics obtained for the 20 biofabricated samples, providing a comparative assessment of their functional and structural properties. The optical transparency and morphological fidelity of the constructs are analyzed.

#### 3.2.1. Transparency

Optical transparency is a critical metric for assessing the functionality of bioprinted corneal scaffolds. According to the literature, the native human cornea exhibits high light transmission, typically ranging from 80% to 94% within the visible spectrum (450–600 nm) and reaching up to 98% at longer wavelengths [17]. Achieving these levels in bioprinted constructs remains a significant challenge in tissue engineering.

In this study, the planar constructs demonstrated high optical transparency, with values ranging from 87.74% to 92.12% across the 450–600 nm range and a maximum of 93.72% at longer wavelengths (see Figure 5E). In contrast, non-planar constructs demonstrated light transmission values ranging from 78.57% to 86.37% across the 450–600 nm wavelength range, reaching a peak transparency of 89.24%.

While these values are slightly lower than those observed in native corneas, they remain within a functional range; in [60], the selected optimal construct with properties most similar to the native corneal tissue showed a light transmittance of 74.7 ± 1.45% at a 500 nm wavelength.

The discrepancy found in the transparency results when comparing planar and non-planar corneas may be attributed to differences in material density and deposition strategy. The proposed method, which utilizes two successive layers of curvilinear trajectories adapted to the anatomical curvature, may have introduced an increase in the material density compared to single-layer planar trajectories. Moreover, non-planar toolpaths result in greater continuity between the extrusion trajectories. While this continuous deposition minimizes the stairstep effect across the geometry, it may simultaneously increase the effective density of the hydrogel, leading to an increased accumulation of material thickness. This increased thickness lengthens the optical path that light must traverse through the material, which inherently elevates internal light absorption and micro-refraction [59]. Consequently, these structural differences may promote light scattering phenomena. This behavior is consistent with experimental observations in nanofibrous scaffolds, where a direct dependence between scaffold thickness and reduced visible light transmission has been reported, attributed to increased optical path length and enhanced scattering within fibrous networks [61]. In contrast, traditional planar slicing tends to produce more fragmented trajectories and an overall less consolidated structure. Although this planar fragmentation compromises surface smoothness, it reduces bioink density and total thickness, which explains its slightly higher light transmittance. Despite this, the non-planar constructs successfully achieved an adequate functional transparency for corneal scaffold applications.

#### 3.2.2. Morphological Fidelity Analysis

**Figure 6:**
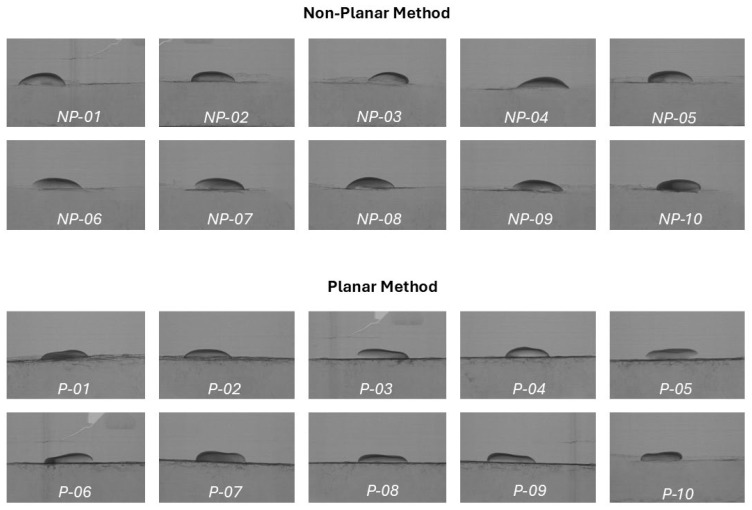
High-resolution cross-sectional projections of 3D-printed corneas captured during the micro-CT acquisition process. The samples are categorized by the printing methodology: the non-planar method (labeled NP-01 to NP-10) and the planar method (labeled P-01 to P-10).

The images captured during the micro-CT acquisition process can be observed in Figure 6. The printed corneas are categorized according to the printing approach: the non-planar method (*n* = 10) and the planar method (*n* = 10). These cross-sectional visualizations provide an immediate qualitative comparison of the structural outcomes. The images corresponding to the non-planar method demonstrate a well-defined corneal structure with a greater vertical elevation in contrast to corneas produced via the planar method, which exhibit a flatter morphology with reduced apical height. These 2D projections serve as visual evidence for the subsequent 3D volumetric reconstructions and quantitative topographic analyses, highlighting the geometric differences between the two methodologies.

##### Height Analysis

**Figure 7:**
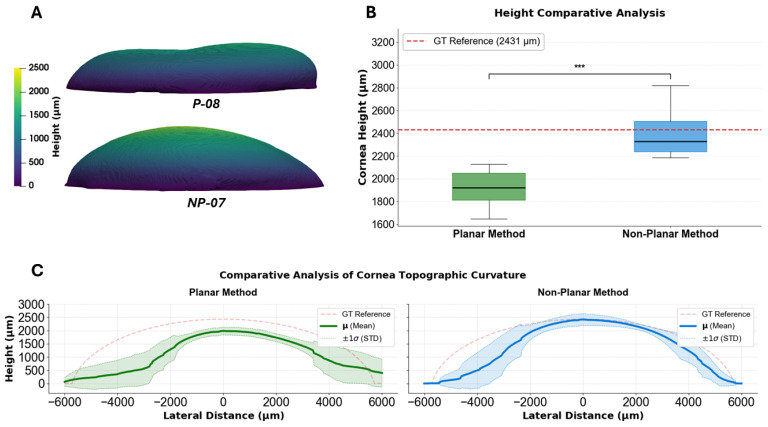
Morphological fidelity analysis results. (**A**) A 3D surface height map of planar (P-08) and non-planar (NP-07) corneal construct, both reconstructed from micro-CT data. (**B**) Comparative analysis of corneal apical height between planar and non-planar printing methodologies (*** *p* ≤ 0.001). (**C**) Comparative topographic curvature profiles of printed corneas. Cross-sectional elevation profiles are plotted against lateral distance. Asterisks (***) indicate statistical significance.

The structural fidelity of both methodologies is evaluated by measuring the maximum apical height across all 20 printed samples. To perform this analysis, a 3D mesh reconstruction is conducted for each specimen, generating a high-resolution height map where surface elevations are represented via a color-coded scalar bar (see Figure 7A).

The quantitative outcomes of these reconstructions are presented as box plots to compare the two printing methodologies (Figure 7B). In this plot, the CAD-designed GT height (2430 µm) is represented by a horizontal dashed line. As illustrated by the resulting distribution, the non-planar method consistently achieved heights closer to the intended design (see Figure 7B), showing a mean height of 2416 ± 219 µm. This corresponds to a deviation of 0.58% from GT, demonstrating high topographic fidelity. In contrast, the planar method exhibited a systemic reduction (20.96%) in height across all the samples, averaging a mean height of 1921 ± 144 µm.

These results demonstrate that the non-planar trajectory yields constructs with superior vertical fidelity, closely approximating the axial dimensions of the original CAD model. The statistical superiority of this approach is validated by an extremely low *p*-value (*p* = 0.00002, ***), confirming that the observed improvements in height reconstruction are highly significant and independent of stochastic variation.

##### Topographic Curvature Profiling

The morphological integrity of the printed corneas is evaluated through curvature profiles. This analysis captures the methodology’s ability to maintain the original smooth parabolic shape from the base to the peak. The results in Figure 7C, categorized by printing methodology, are compared against the CAD model. The curvatures achieved with the non-planar method (blue) show a high degree of resemblance to the GT curve, significantly outperforming those obtained with the planar method (green). This superiority is further quantified by the AUC, where the non-planar method achieved a topographic fidelity of 77.24% relative to the GT. The standard deviation remains narrow, indicating the reproducibility of the curvature in both cases; however, in the case of planar curvatures, a flattened result is obtained, failing to reach the target elevation of the peak and the curvature shape. In the planar method, the AUC fidelity drops to 60.17%, confirming that the geometric deviation of the planar method is not localized to the peak but represents a systemic structural collapse of the intended curvature across the entire structure. Consequently, the non-planar approach proves essential not only for curvature accuracy but also for preserving the integrity required for a functional topographic reconstruction.

## 4. Conclusions

The present study successfully developed and validated a novel non-planar trajectory generation framework for corneal biofabrication. The integration of the Poisson mesh reconstruction and surface parametrization proved to be a robust method for translating 2D toolpath patterns onto complex 3D curvilinear geometries. This approach effectively bridges the gap between traditional CAD slicing and the high-precision requirements of thin-film ocular tissues. Importantly, from a manufacturing perspective, the computational overhead is limited to a brief initial configuration phase. The optimized G-code can subsequently be executed indefinitely to mass-fabricate scaffolds without incurring repetitive processing delays.

The quantitative analysis of the bioprinted corneal constructs demonstrated that the non-planar methodology significantly outperformed traditional planar layering in maintaining anatomical proportions. The non-planar constructs achieved an apical height closer to the target (CAD corneal model) and a similar curvature profile, whereas planar samples suffered from a loss of height. By allowing the extrusion printhead to follow the natural curvature of the model, the non-planar approach minimizes the stairstep effect typically observed in layer-by-layer deposition. The trajectories generated with the proposed approach enable a smoother surface finish and a more stable dome, which suggests improvements in the mechanical stability of a corneal scaffold. Although the light transmission values recorded for corneal constructs printed with the proposed methodology were lower than those obtained using the planar methodology, they remain within an acceptable functional range and may be attributed to differences in material density and deposition strategy.

Furthermore, this study addresses a critical gap in the existing literature by systematically reporting measured geometric errors as a function of curvature. By analyzing curvature variations and their corresponding dimensional deviations using high-resolution scanning, this study provides the empirical data necessary to bridge the gap between digital design and biofabrication. Such measurements offer concrete guidance for future scaffold optimization and help establish uniform evaluation criteria, which will facilitate meaningful comparisons across studies and accelerate the translation of 3D-printed corneal constructs into clinical applications.

Moving forward, research should focus on balancing the trade-off between mechanical enhancement and optical transparency, while exploring the potential of bioprinting multi-layered structures, such as the epithelium and stroma. For stromal biofabrication, this method aligns closely with native tissue structures. The literature emphasizes that the outermost 100–120 µm of the anterior stroma consists of a tightly interwoven, undulating collagen network responsible for maintaining corneal curvature [16]. Utilizing non-planar trajectories might replicate this curved macro-architecture. The non-planar environment might provide a more physiologically representative architecture for cellular organization, which may crucially influence spatial cell positioning and native intercellular interactions. This stands in contrast to flat, concentric printing, which can induce highly restrictive contact-guidance tracks—grooves left by the printing nozzle that physically force encapsulated cells to align and migrate along artificial, unidirectional circular pathways [62]. By minimizing the stairstep defects characteristic of traditional flat slicing, this method yields a continuous, smooth surface finish. This curvature is intended to provide an anatomically representative basement layer for uniform epithelial cell seeding, while simultaneously aiming for adequate functional optical quality by reducing potential light scattering.

## Data Availability

Data are available upon request from the corresponding author. The data are not publicly available because the original contributions presented in this study are included in the article.

## Abbreviations

The following abbreviations are used in this manuscript:

AM	Additive Manufacturing
AUC	Area Under the Curve
CAD	Computer-Aided Design
DICOM	Digital Imaging and Communications in Medicine
DLP	Digital Light Processing
GT	Ground Truth
Micro-CT	Micro-Computed Tomography
PBS	Phosphate-Buffered Saline
ROI	Region of Interest
STL	Stereolithography Interface Format
